# General Purpose Alarm Device: A programmable annunciator

**DOI:** 10.1016/j.ohx.2024.e00590

**Published:** 2024-10-10

**Authors:** Robert L. Read, Lawrence Kincheloe, Forrest Lee Erickson

**Affiliations:** a Public Invention, 1709 Norris Dr., Austin, TX, 78704, United States of America; b SPEC & Public Invention, 3217 Brush Creek Road, Oklahoma City, OK 73120, United States of America

**Keywords:** Alarms, Arduino, Uno, SPI, Medical alarms, Monitoring

## Abstract

The General Purpose Alarm Device (GPAD) shines lights and makes loud noises to draw the attention of a human being to a problem. It provides a programmable, 80 character display to provide textual information. As an inexpensive modular annunciator, it is intended to decrease the cost of any system that requires complex monitoring and rapid human intervention. Fundamentally, it is designed to act as a peripheral to a controlling computer or microcontroller. The controller may communicate over a USB (COM) connection or a 5V SPI connection via an RJ12 cable. The GPAD is intended to be as general purpose as possible, so that it can be used to provide alarm functionality for many engineering and scientific projects, hobby machines, instruments, and various situations. The original driving use case is to provide medical alarm capability to the PolyVent open-source mechanical medical ventilator. The GPAD supports 5 alarm levels above “silent” of increasing urgency in terms of light, rhythm, and frequency. It has a mute button. It is based on the Arduino Uno Atmega328 design and is potentially extensible through headers and shields like an Uno. The GPAD includes a printed wiring assembly, firmware for the GPAD peripheral, a simple documented API and a 3D printable enclosure. The repo includes instructions for using a second GPAD as a controller as an example for programming.

## Graphical abstract

**Figure:**
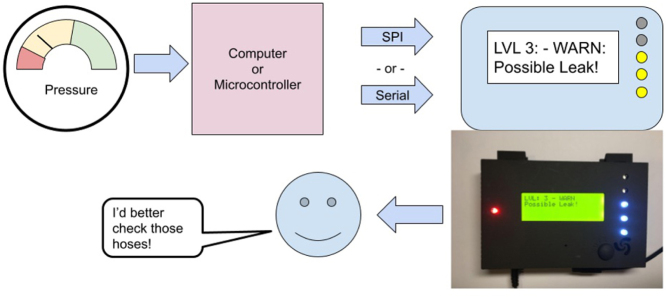

**Table:** 

**Specifications table**	

Hardware name	General Purpose Alarm Device (GPAD) v2.0
Subject area	General
Hardware type	Other: general alarms for digitally detectable alert conditions
Closest commercial analog	No known commercial analog is available
Open source licence	*CERN Open Hardware Licence Version 2 - Strongly Reciprocal*
Cost of hardware	$100.00
Source file repository	https://zenodo.org/records/13825787 and GitHub repository [1].
OSHWA certification UID	US002352

## 1 Hardware in context

Fire safety and medical alarms are typically organized into complete systems which must be treated and tested holistically [2]. This work specifically addresses only the “alerting” or “annunciation” part of the problem of building a complete alarm system. Our goal is to make a reusable module [1] that can be generally used in a wide variety of systems that must alert humans to problems requiring rapid human response. For example, in terms of a complete automated health monitoring system [3], the GPAD would be considered an “edge device”. In fact, the original motivation for the GPAD was to act as the alarm device for the PolyVent [4] mechanical ventilator. Although GPAD can be placed far from the system that it is monitoring, for example in a nurse’s station on a multi-bed clinic ward, it is a programmable IoT edge device for obtaining human attention. It could therefore be part of a simple modular component of clinic-wide monitoring system, such as for monitoring medical gases [5].

We are not aware of any alarm device that has the programmable features of the GPAD. Electronic component firms such as Mouser and Digikey sell buzzers, sirens, loudspeakers which can make noise, lights that can represent an alarm condition, and proprietary alarm devices that are tied to special purposes (such as a smoke alarm). However, we know of no digitally controllable system that combines these features. The Adafruit Towerlight [6] is an example of a “stacklight”, but it does not seem to have a digital interface, a text output capability, or an abstract API. Mucco makes a complete line [7] of integrated warning horns and stack lights, but they appear to have no digital interface, text, or alarm levels, and instead are simply on or off. Although we believe a GPAD is a widely useable device, its use is confined to manufacturers, makers, or engineers who are capable of programming it, which may preclude the market volume associated with consumer devices.

Digital controllers for 3D printers can often beep and have a digital display, which is sometimes backlit. This has some of the features of the GPAD, but is not as bright or as loud, nor programmed for this purpose as conveniently. A cheap example is the HiLetGo 3-01-0889 [8].

The GPAD is potentially advantageous for research laboratories that need to alert researchers to a wide variety of conditions and problems, whereas a simple non-programmable annunciator (like a horn, buzzer, or stacklight) would not provide enough information. Although the GPAD requires some very modest programming skill to utilize, it offers informative annunciation and an 80-character text display that can powerfully inform the operator regarding a complex condition. This applies to medical and industrial situations as well, but research laboratories may have the most *ad hoc* error conditions of any situation.

## 2 Hardware description

We have designed a printed wiring assembly and an enclosure. The enclosure is derived from the parametric OpenSCAD project “The Ultimate box maker” [9]. The enclosure is approximately 160 mm x 110 mm x 50 mm (6${}^{\prime \prime }$ × 4.25${}^{\prime \prime }$ × 1.75${}^{\prime \prime }$). The assembly weighs 232 grams (without any power supply). The device has a 20 × 4 character display (ASCII English characters), five bright white programmable LEDs, a programmable buzzer, a “Mute” button which silences the buzzer and a non programmable power indicator LED. The enclosure hangs by (for example) tie wraps from a rail for vertical display, or can rest on any horizontal surface. Typical signal connection is via an RJ12 data cable. Power is provided by an external wall supply with a 2.1 mm barrel jack power connector at 9–12 V at less than 1000 mA.

The GPAD integrates several features that hobbyists may find useful into one device. Although you can purchase 20 × 4 displays with parallel or I2C interfaces, it is the integration in one PCB and enclosure of all of these features which makes this tethered device integrate into other systems easily.

An end-user will interact with the following GPAD hardware features:

• Five bright LEDs to visually indicate alert levels.
• A buzzer to indicate urgent attention is required.
• A button for minimum user feedback and firmware toggles, which turns the audio alarm on or off.
• A backlit 20 × 4 character display to provide alarm detail to the user.
• An enclosure that can be vertically mounted by hanging with wire ties, for example, or placed horizontally.

**Example Use Case**

Scientific experiments and laboratory equipment often monitor something over time whose condition can reach a state that requires human attention. Thus, the GPAD is useful in the field or laboratory for:

• Alerting to conditions which may ruin the experiment if not corrected.
• Alerting to the very conditions sought by the experimenter and therefore demanding attention.
• Indicating potential failure of the equipment or the need for maintenance, such as low battery charge, over-temperature, over-pressure, etc.

The GPAD is designed to be loosely integrated with new devices. At the cost of modifying the enclosure, a researcher may integrate the GPAD into their own equipment. Such an integration would likely be a premature optimization for most research labs, but might be economically justified if more than a hundred systems requiring monitoring were being constructed.

## 3 Design files summary

Design files are found at the Public Invention Zenodo repository at: https://zenodo.org/records/13825787.

**Table:** 

Design filename	File type	Open source licence	Location of the file(s)
Firmware	A folder with source files. A README.md there describes the various source files	Firmware: Affero GPL 3.0	https://zenodo.org/records/13825787/preview/general-purpose-alarm-device-2.0.2.zip?include_deleted=0#tree_item3

Simulation	Wokwi, simulations for Factory Test and other development efforts.	Firmware: Affero GPL 3.0	https://zenodo.org/records/13825787/preview/general-purpose-alarm-device-2.0.2.zip?include_deleted=0#tree_item635.

Hardware	Folder holding a README describing the subfolders to follow.	CERN Open Hardware Licence Version 2 - Strongly Reciprocal	https://zenodo.org/records/13825787/preview/general-purpose-alarm-device-2.0.2.zip?include_deleted=0#tree_item98

GeneralPurpos eAlarmDevicePCB	KiCad schematic and PCB files	CERN Open Hardware Licence Version 2 - Strongly Reciprocal	https://zenodo.org/records/13825787/preview/general-purpose-alarm-device-2.0.2.zip?include_deleted=0#tree_item177

Enclosure	OpenSCAD and FreeCAD source files, STL and STEP files for the enclosure	CERN Open Hardware Licence Version 2 - Strongly Reciprocal	https://zenodo.org/records/13825787/preview/general-purpose-alarm-device-2.0.2.zip?include_deleted=0#tree_item151

Manufacturing sub assemblies	bill of materials, Gerber files and description records of parts ordered for the builds.	CERN Open Hardware Licence Version 2 - Strongly Reciprocal	https://zenodo.org/records/13825787/preview/general-purpose-alarm-device-2.0.2.zip?include_deleted=0#tree_item177

Final assembly manufacturing and unit test documentation	Assembly instructions and notes	CERN Open Hardware Licence Version 2 - Strongly Reciprocal	https://zenodo.org/records/13825787/preview/general-purpose-alarm-device-2.0.2.zip?include_deleted=0#tree_item99 file: ManufacturingUnitTestTroubleshootingRev2.md.

The main firmware directory contains a regression test plan and the following directories:

• The GPAD_API directory holds the main code.
• The GPAD_API_SPI_CONTROLLER directory contains code to use the GPAD itself as a test controller for the GPAD (requires two).
• GPAD_Factory_Test contains a run through of basic hardware to make sure the GPAD is correctly assembled.
• The directory named “simulation” contains files for our WokWi simulation.
• The directory “Hardware” contains the Schematic and PCB KiCad design files, our KiCad library files, and a “Manufacturing README.md” for the printed circuit assembly and other manufacturing documentation.
• GeneralPurposeAlarmDevicePCB contains KiCad PCB design source files, Gerber Files, and Parts lists.
• Enclosure contains the FreeCAD source and generated .STL files for 3D printing the plastic enclosure.
• Manufacturing contains special files used in our order to JLCPCB, a PCB assembly manufacturing house.
• Documentation/ManufacturingUnitTestTroubleshootingRev2.md contains instructions and photographs of assembling a complete GPAD, similar to the instructions included in this paper.

## Bill of materials

The spread sheet used to receive the KiCad generated BOM/PARTS LIST and to transform it into the files necessary for ordering PCB, assemblies from JLCPCB are found at: https://zenodo.org/records/13825787/preview/general-purpose-alarm-device-2.0.2.zip?include_deleted=0#tree_item177 under file GPAD_V2_BOM.xls.

The spreadsheet contains built in instructions for its use. JLCPCB built the raw PCB, performed the surface-mount device (SMD) assembly, and some through-hole assembly. The balance of the through-hole assembly was done by Public Invention volunteers.

## 4 Bill of materials summary

**Table:** 

Designator	Component	Number	Cost per unit - currency	Total cost - currency	Source of materials	Material type
Enclosure LED standoff	U_Box_V105_GPAD _LED_Standoff_single.stl	6	$1.01	$1.01	Fabricated by JLCPCB	321PA–F NYLON

Enclosure frontpanel	U_Box_V105_General_Alar m_Device_FrontPanel.stl	1	$3.05	$3.05	Fabricated by JLCPCB	321PA–F NYLON

Enclosure Top	U_Box_V105_General_Alar m_Device_Top.stl	1	$12.4840	$12.48	Fabricated by JLCPCB	321PA–F NYLON

Enclosure bottom	U_Box_V105_General_Alar m_Device_bottom.stl	1	$13.00	$13.00	Fabricated by JLCPCB	321PA–F NYLON

Enclosure button	U_Box_V104_General_Alar m_Device_button.stl	1	$1.00	$1.00	Fabricated by JLCPCB	321PA–F NYLON

Enclosure backpanel	U_Box_V105_General_Alar m_Device_Backpanel.stl	1 NOTE use of this is exclusive of the BackPanel Hanging Compound	$3.34	$3.34	Fabricated by JLCPCB	321PA–F NYLON

Enclosure BackPanel_Hanging	BackPanel_Hanging–CompoundV2.stl.	1 NOTE use of this is exclusive of the BackPanel	$5.90	$5.90	Fabricated by JLCPCB	321PA–F NYLON

Enclosure screws PCB mounting	Choose to fit the enclosure as fabricated	5	$0.10	$0.50	Used #6 sheet metal screws 3/8” long	Steel

Enclosure screws,	Choose to fit the enclosure as fabricated	4	$0.10	$0.40		Steel

PCB assembly exclusive of through hole components	BOM_JLCPCB_20230228 Modified.xls	1	$13.86	$13.86	Fabricated by JLCPCB	GeneralPurpose AlarmDevicePCB–Placement20230 228Modified.xls

U302	LCD Display	1	$4.99	$4.99	https://www.aliexpress.com/item/3256803213374992.html	3256 Display Assembly

Nylon spacers	Spacer_0.0182 × 0.125 inch	4	$0.13	$0.52	McMasterCarr	94639a702

Screw	4–40x 3/8”	4	$0.12	$0.48	Digikey	36–9901–ND

Nut	Nut_4–40_3/16	4	$0.10	$0.40	Digikey	36–4694–ND

S101	SWITCH_TACTILE_SPST-NO_0.05A_24V	1	$0.13	$0.13	Digikey	450–1804–ND

S601	SWITCH_TACTILE_12m mx12mm_SPST–NO_0.05A_24V	1	$0.55	$0.55	Digikey	SW414–ND

LED white	LED_T1.75_CLEAR_WHITE	6	$0.65	$3.90	Digikey	160–1772–ND

LED red	LED_T1.75_CLEAR_RED	1	$0.36	$0.36	Digikey	160–1682–ND

RV301	POT 0.375 10 K	1	$1.61	$1.61	Digikey	3386P–103LF–ND

9–12 V wall power supply	Example Jameco.com 9 V 200 mA	1	$6.95	$6.95	Jameco.com	10 0845

These parts sum to $74.43 in 2023.

## 5 Build instructions

Partial assembly of the GPAD consists in PCB fabrication. Most components are from JLCPCB. Components not available at JLCPCB were later added by Public Invention.

The printed wiring assemblies were ordered from JLCPCB using a BOM file and a placement file:

• https://zenodo.org/records/13825787/preview/general-purpose-alarm-device-2.0.2.zip?include_deleted=0#tree_item341 in files BOM_JLCPCB_20230228Modified.xls and GeneralPurposeAlarmDevicePCB-Placement20230228Modified.xls.

LED stand-offs were 3D printed from JLCPCB.

Order six for each assembly, or for each LED, with file:

• https://zenodo.org/records/13825787/preview/general-purpose-alarm-device-2.0.2.zip?include_deleted=0#tree_item151 file U_Box_V105_GPAD_LED_Standoff_single.stl.

Enclosures were ordered 3D printed from JLCPCB.

To build an enclosure, print each of these six files:

• File BackPanel_Hanging-CompoundV2.stl,
• Files U_Box_V105_General_Alarm_Device_Backpanel.stl,
• U_Box_V105_General_Alarm_Device_FrontPanel.stl,
• U_Box_V105_General_Alarm_Device_Top.stl,
• U_Box_V105_General_Alarm_Device_bottom.stl, and
• U_Box_V104_General_Alarm_Device_button.stl

from folder (and subfolders): https://zenodo.org/records/13825787/preview/general-purpose-alarm-device-2.0.2.zip? include_deleted=0#tree_item151.

The time to build assemblies (40 min) is documented in BuildTime.md.

Detailed instructions for the remainder of the manufacturing steps was developed by journaling work on the assemblies received from JLCPCB. The journal is captured in a markdown document, “Manufacturing and Unit Test Documentation, PCB Version 2.0, 20230228” found at: https://zenodo.org/records/13825787/preview/general-purpose-alarm-device-2.0.2.zip?include_deleted=0#tree_item99 in file ManufacturingUnitTest TroubleshootingRev2.md.

Manufacturing and Unit Test Documentation, PCB Version 2.0 February 28, 2023.

**Tools Required**

**Fig. 1:**
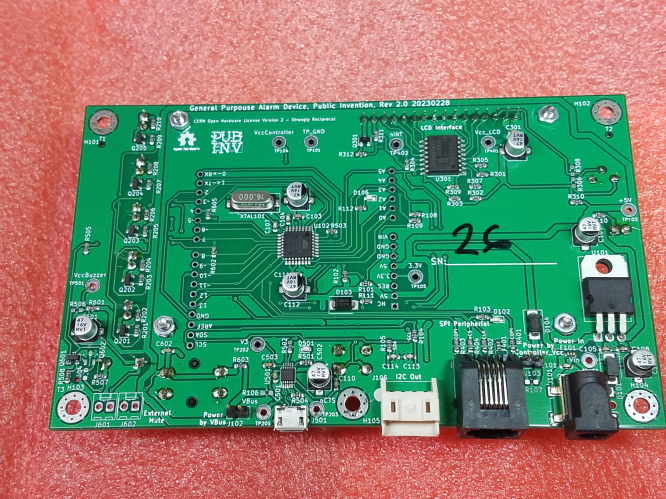
The PCB assembly received from JLCPCB. Note Serial #26 hand-written on PCB.

**Fig. 2:**
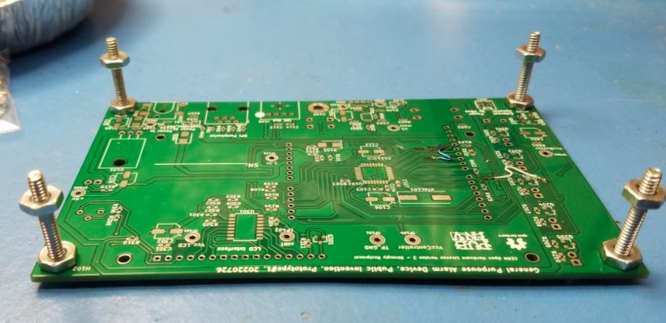
Required rework for GPAD Rev 1.x PCB.

• Solder station with appropriate ventilation.
• 3/16 Nut Driver.
• Number 1 Phillips screw driver.
• Diagonal or other flush cutting hand tool for lead trimming.
• Assembly fixture, detailed in Fig. 2.

**Assembly Reference Material**

The most recent schematic for Rev 2 PCB Assemblies can be found in PDF form here: https://zenodo.org/records/13825787/preview/general-purpose-alarm-device-2.0.2.zip?include_deleted=0#tree_item237 in file Schematic-GeneralPurpose AlarmDevicePCB-V2.2.pdf.

**Assembly Notes and Tips**

• The board will be received from JLCPCB with SMT components placed and some, but not all, through-hole components. See Fig. 1.
• The serial number should be written on the PCB assembly at the location provided. See Fig. 1.

Further management of serial numbers is beyond the scope of this document.

**An Assembly Fixture**

• An assembly assistant/fixture can be made by using a raw PCB with some long #6 screws and nuts to hold at the PCB mounting points. Refer to Fig. 2.

***Please note:*** *The following changes have been made to the GPAD Rev 2.0 PCB per issue #213:*

• *Resistor R103 has been removed.*
• *Factory test for D601 has been updated.*

*The problem that was identified is that the assemblies built per the BOM_JLCPCB_20230228Modified.xls have R103 fitted with a 1 K resistor. This resistor, together with LED D102, loads the SPI_CLK signal and is incompatible with proper operation of the GPAD as an SPI Peripheral from a 3.3 V SPI Controller using the level shifting method and the common gate MOSFET.*

**Fig. 3:**
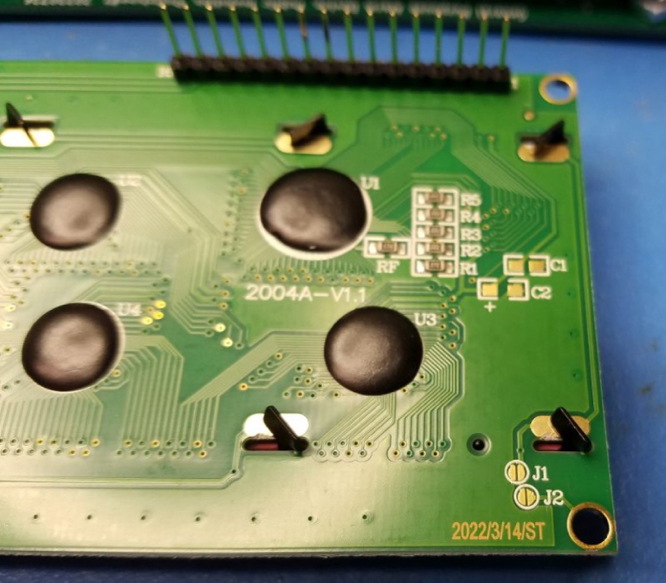
The LCD Module rear view, header placed.

**Fig. 4:**
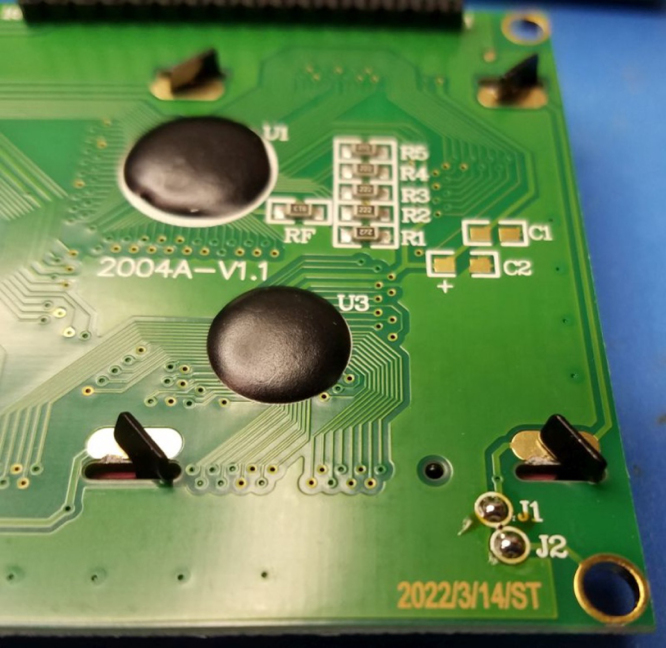
Ground Bezel of J1, J2.

*The recommended solution is to remove R103.*

## Assembly steps

**Fig. 5:**
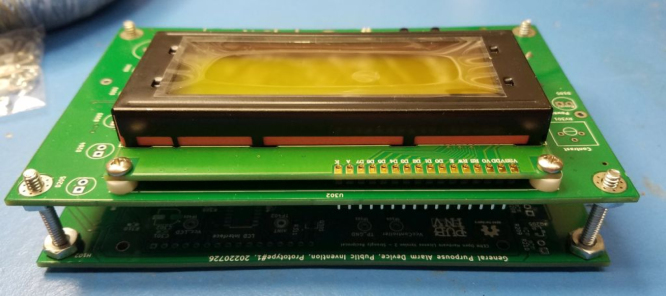
The LCD Module assembled to the PCB, screws, spacers, and header.

**Fig. 6:**
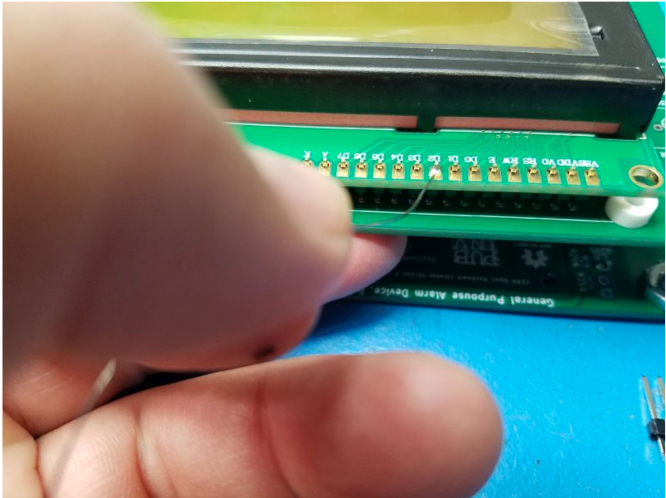
Solder the header pin on the display.

**Fig. 7:**
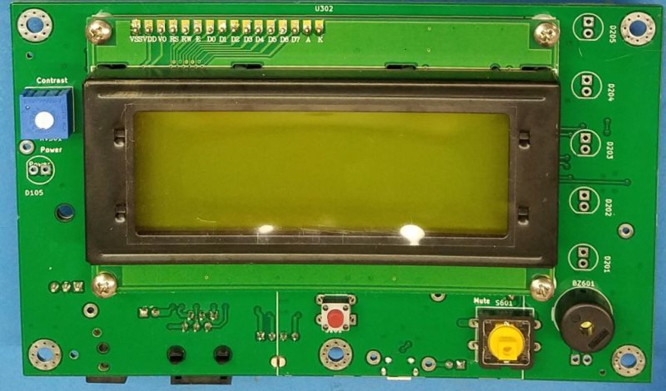
Location of RV310, 5101, 5401, B2601.

**Fig. 8:**
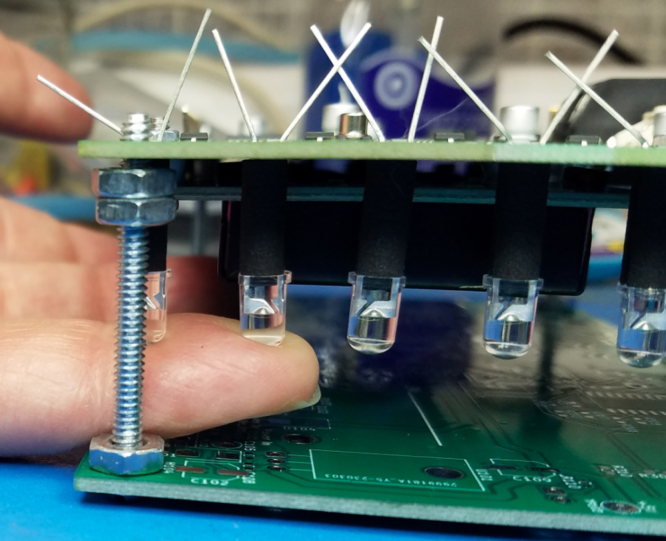
Installation of LEDs, and spacers with leads bent to hold in place.

1. **LCD Bezel Grounding**
  • Locate the J1 and J2 solder pads on the LCD module at the bottom right as shown in Fig. 3.
  • Solder them, after which they will appear as in Fig. 4.
2. **16-pin Header**
  • Fit the 16-pin header onto the LCD sub-module on the top of Fig. 3.
  • Solder the header pins to the display (see Fig. 6). Solder one pin, and with your free hand, reheat the solder, then lift the header flush to the board and remove heat from the pin. Solder the rest of the pins.
  • Place four nylon 1/8${}^{\prime \prime }$ spacers at the four corners of the LCD sub-module.
  • Place four 4-40 x 3/8${}^{\prime \prime }$ screws with 4-40 x 3/16${}^{\prime \prime }$ nuts through both boards and torque to 3.4 - 4.8 Inch-Pounds.
  • Solder the header pins to the GPAD PCB.
3. **Buttons and Potentiometer** (**Refer to** Fig. 7)
  • Place the Reset button, S101, into the PCB from the display side.
  • Place the Mute button, S401, into the PCB from the display side.
  • Place the Buzzer, BZ601, into the PCB from the display side. Bending the leads may help keep it in place.
  • Place the Contrast pot, RV301, into the PCB from the display side.
  • Solder to PCB.
4. **LEDs (Refer to** Fig. 8 **)**
  • Thread the LED leads through the stand-offs.
  • The longer LED lead is the anode. The cathode has a flat side on the plastic case.
  • Place the LEDs into the PCB so that the flat cathode side corresponds to the silk screen marking. Bend the leads to retain the LED into the PCB.
  • Placing the assembly on the fixture lets you have access to the top and the bottom of the assembly (see Fig. 5)*.
  • Lift the LED with a finger and reheat solder for a flush fit on the PCB.
  • For each LED, solder one lead by holding the LED from below supported by the spacer, and hold against the PCB. Then, reheat the solder for a flush fit.
  • Solder the second lead on the LED.
  • Trim the excess leads on RV301 and the LEDs.

**Assembly Tip:** Sharpie Oil-Based Paint Markers can be used to mark polarity on LED standoffs and mark PCB’s version, serial number and the programming status of the microprocessor.

*Congratulations! Electrical assembly is complete.*

## Electrical tests

Electrical tests are divided into two parts: 1. Unprogrammed measurements made before flash programming the boot loader and other firmware and 2. Programmed measurements made after a boot loader and firmware have been placed into the microcontroller.

**Electrical Measurements before programming bootloader and firmware**

Measure and record in Table 1 (included as an example) the following electrical parameters by serial number.

Investigate and correct any abnormal measurements before applying power. Remove J102 and J103 and retain them if present. Note where they should be replaced. Start with no connections to the DUT (Device Under Test).

• **Power Jack:** Measure resistance to ground at J101 centre pin as open or greater than 1Meg ohms.
• **SPI Interface** Measure resistance to ground at J401 pin 5 as open or greater than 1Meg ohms.
• **Vin net:** Measure resistance to ground at TP102 as greater than 75 K ohms. This net is capacitive and the resistance measured will climb as the multimeter charges the net.
• **+5V net:** Measure resistance to ground at TP103 ＋5 at 1 K +/−5% (950–1050 Ohms).

After having measured and recorded those resistances, use a current-limited supply configured for 12 V and a maximum of 200 mA and apply power at J101. Measure:

• **Unprogrammed Current**: Note and record the unprogrammed current. (FYI, when unprogrammed, the first-time power up current is normally about 75–80 mA.)
• **LED Check:** Check that the power LED D105 is lit and is RED.
• **+5V net:** Measure the voltage of the +5V Net at TP103.

Please be advised that the current drawn by a programmed DUT that has been powered up, with a display backlight on, is approximately 61 mA when the reset switch is held down.

***Vo Initial Set/LCD Contrast.***

With a voltmeter, measure the voltage of the Vo pin of the LCD header to ground. Adjust RV103 for 1.3 V.

See records of measurements of some of the Rev 2 assemblies at this url: https://zenodo.org/records/13825787/preview/general-purpose-alarm-device-2.0.2.zip?include_deleted=0#tree_item99 in file: Range_of_V0_for_LCD_Contrast.

**Example Electrical Test Results Table**

Add rows to the record Table 1 for each device under test.

Capture: DUT Serial Number, R@PowerJack, R@SPI Interface, R@Vin net, R@5V net, UnProgramCurrent, Volt@+5 TP103, FullCurrent mA, Vo Volts, and Notes.

Apply power and measurements of current within subcircuits (Issue #230). See actual data from Rev2 build in file Rev2BuildCurrentOnCircuitBlocks here: https://zenodo.org/records/13825787/preview/general-purpose-alarm-device-2.0.2.zip?include_deleted=0#tree_item99. Test condition. Measure before firmware is loaded into device

**Table 1:** A table for recording electrical tests for each DUT.

DUT serial number	R@Powe rJack	R@SPI Interface	R@Vin net	R@5V net	UnProgr amCurrent	Volt@+5 TP103	FullCurr ent mA	Vo Volts	Notes
									

**Capture current on circuit blocks into**Table 2.

These measurements were made before the DUT was programmed with any firmware, even NO BOOTLOADER. Measure the current by measuring the voltage across the 1 Ohm decoupling resistor from the raw power to the test points indicated. Raw power is at TP103 ＋5 test point. Volts in mV will be a measurement of mA.

**Table 2:** Measurements before DUT programmed.

DUT S#	VR101(Current U102) mA	VR310(Current LCD) mA	VR601(Current Buzzer) mA	Notes
29	2.1	49.1		
30	2.5	48.8		

*End of tests with firmware. Now go onto loading firmware.*

## Load firmware

***Load Bootloader***

Use an Arduino Uno as an ISP (In Circuit Serial Programmer) which will load the boot loader into the DUT. Cable connect the ISP Uno to the DUT as follows:

**Wiring of the Uno to the DUT**

**Fig. 9:**
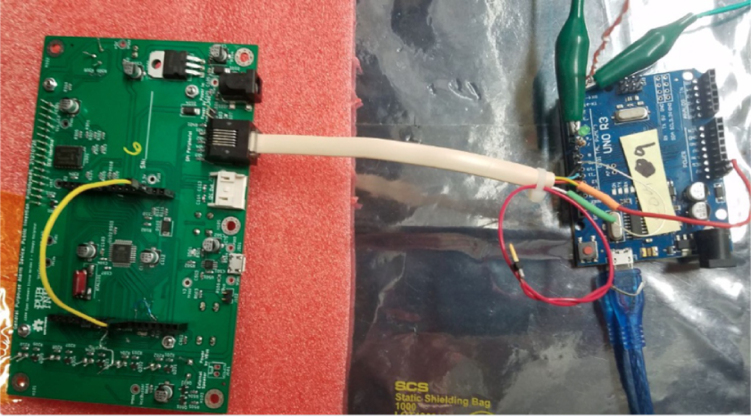
Unit under test (left) and Uno as programmer (right).

**Fig. 10:**
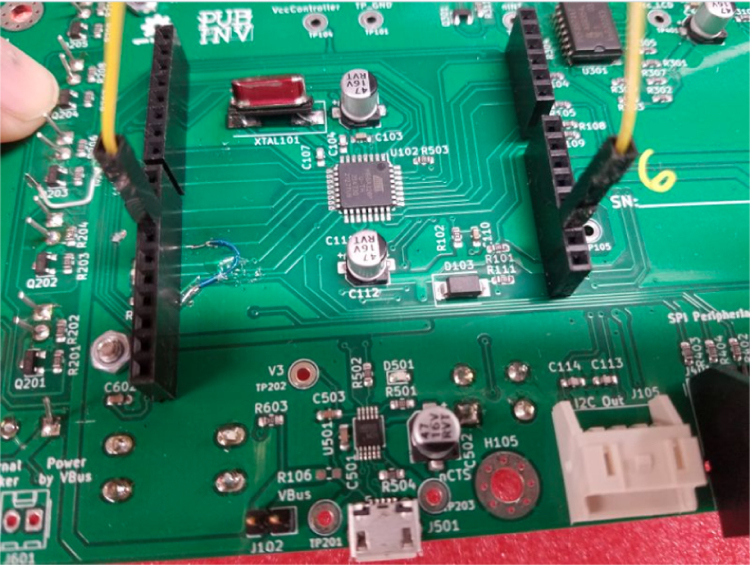
Detail shows the point of view from D10 to reset signals.

Please observe in Fig. 9 an Uno connected to an ISP bootloader into a GPAD through the RJ12 (see Fig. 10).

Place a Jumper on the DUT from D10 to Reset Jumper for D10 to Reset.

**Missing Jumper to D10 When Loading the Bootloader**

If you forget the above jumper the IDE will give an error something like this:



Double check connections and try again, or use -F to override this check.

Error while attempting burning of bootloader without the RJ12 connection.

This report would have more information with the “Show verbose output during compilation” option enabled in File -&gt; Preferences.

***Prepare the Arduino IDE to be an ISP***

**Fig. 11:**
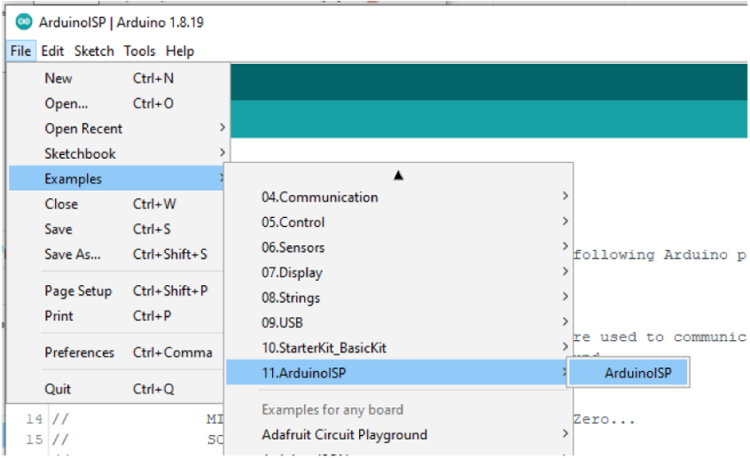
In Circuit Serial Programmer Setup.

To load into the Uno, Select the serial port for the Uno and compile and upload with the ArduinoISP by pressing CTRL **U** as shown in Fig. 11.

**Fig. 12:**
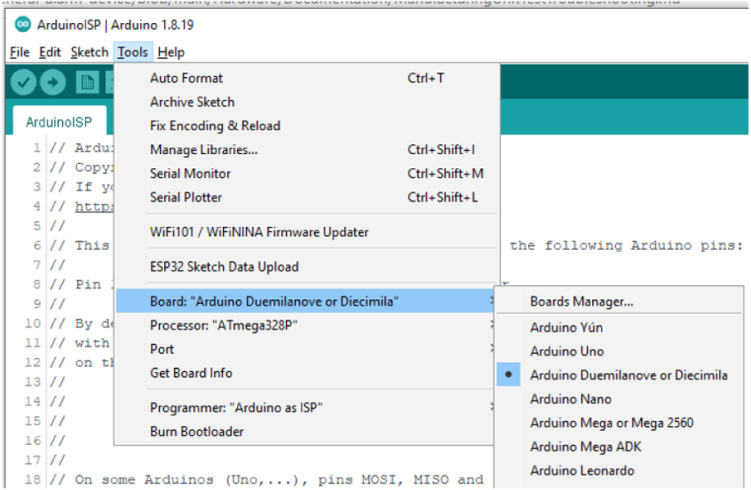
Selecting Duemilanove.

Setup the Uno to burn the boot loader into the GPAD target. Select the board type (Boot loader type) to Arduino Duemilanove\ldots as shown in Fig. 12.

Select the Processor type to ATmega328P as per Fig. 12.

**Fig. 13:**
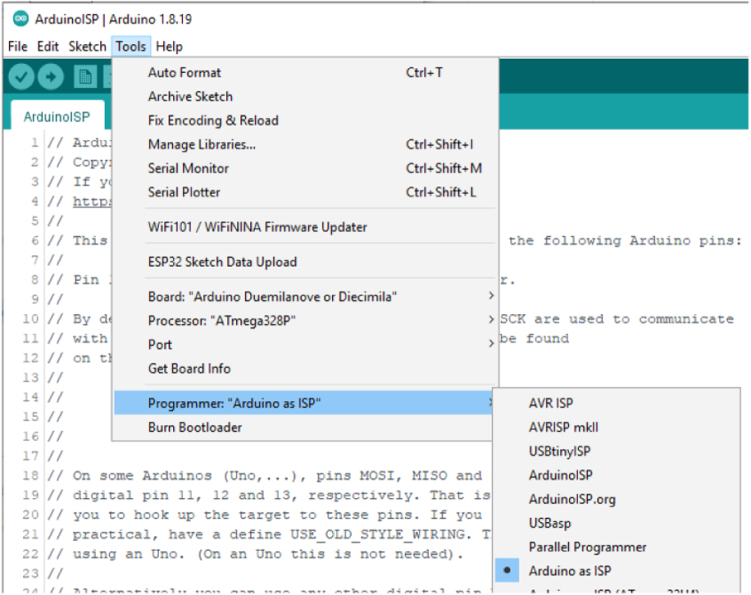
Using the ISP.

Select the programmer type: Arduino as ISP as shown in Fig. 13.

**Fig. 14:**
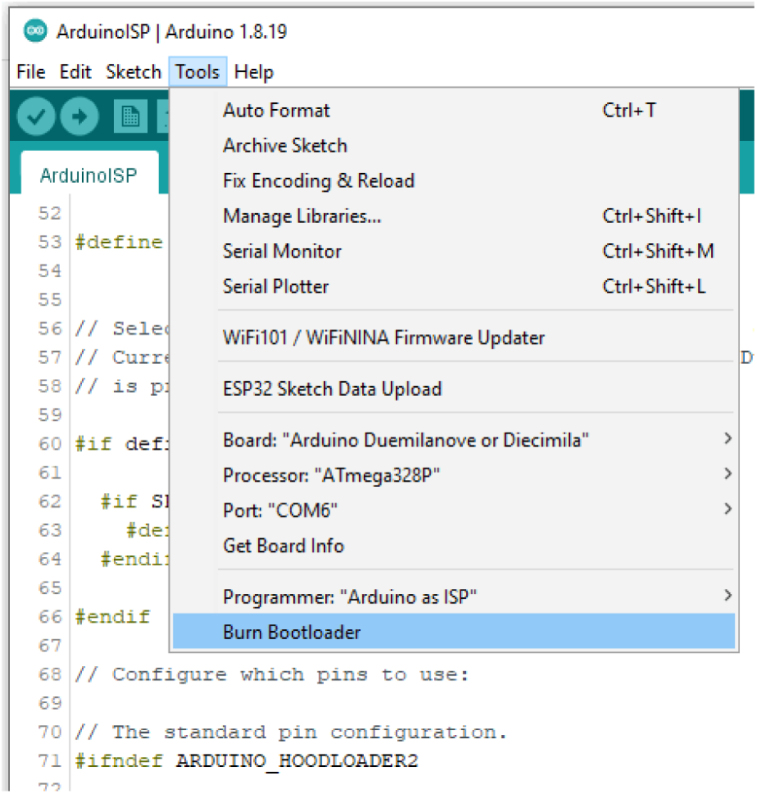
Burning the Bootloader.

In the Arduino IDE, select TOOLS -&gt; Burn Bootloader as shown in Fig. 14.

**Fig. 15:**
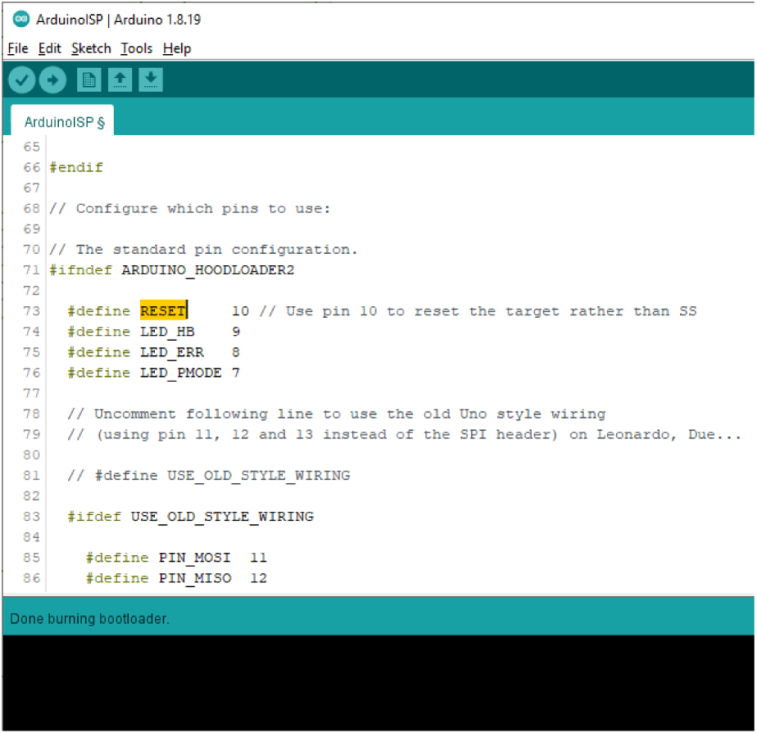
Message Confirming “Done Loading Bootloader”.

Watch the progress bar in the IDE and look for success with the message Done burning bootloader in the blue status bar (See Fig. 15).

If R103 has not been removed, check that LED D102 is winking with a short “on time” and longer “off time”, indicating that the boot loader has been loaded. Until any other sketch is loaded, this is the expected behaviour of the unit under test. Now remove R103.

***Load Factory Test Firmware (sketch)***

Connect a USB cable to the DUT. Note the COM port enumerated in Device Manager Ports(COM&LPT) drop down.

**Fig. 16:**
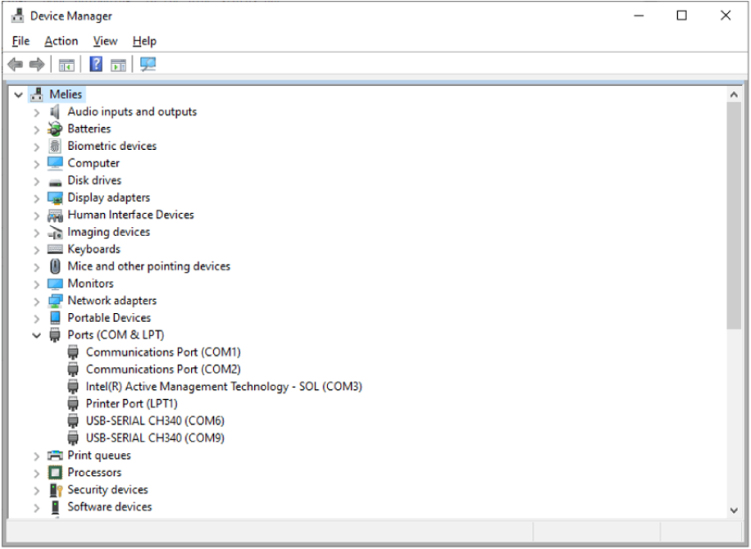
Selecting COM Ports.

In the Arduino IDE, open the new file “GPAD_Factory_Test.ino”. Set the IDE for the COM port of the DUT as exemplified in Fig. 16. Using the Arduino IDE, compile and upload to the DUT the “GPAD_Factory_Test.ino”

**Fig. 17:**
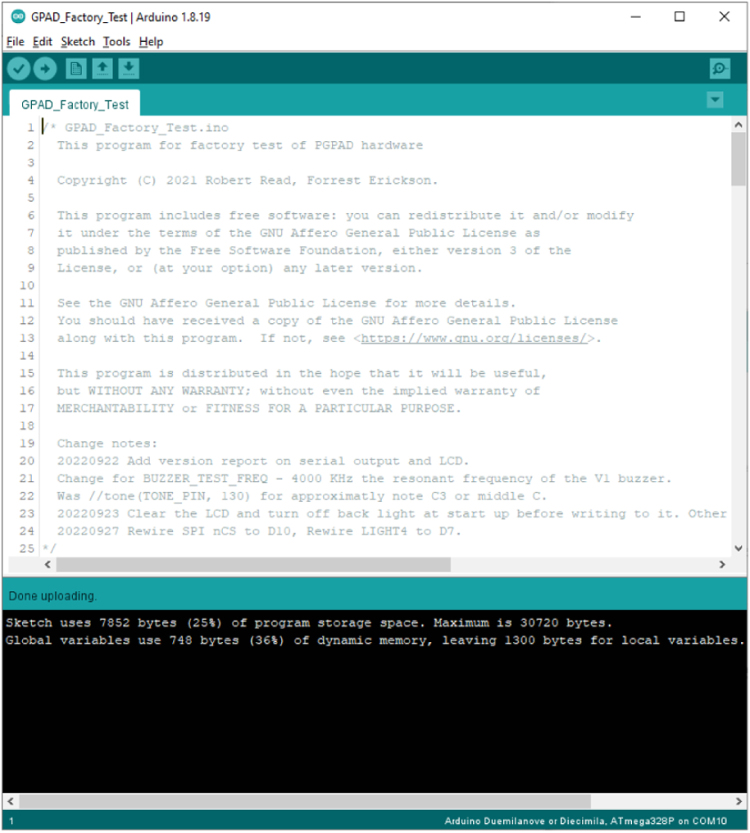
Confirming Load of GPAD_Factory_Tost sketch.

Watch the progress bar in the IDE and look for success with the message “Done uploading” in the blue status bar as shown in Fig. 17.

**Fig. 18:**
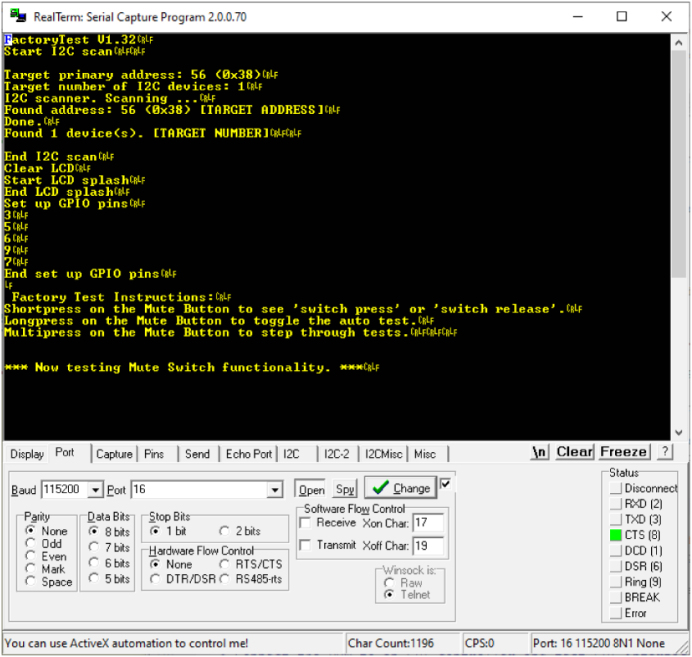
Using a Terminal Program for Testing.

Open a terminal to the COM port of the DUT and set for appropriate BAUD rate 115200. Press the reset switch on the DUT and the LCD should display a message. The terminal should display a boot message too. Fig. 18 is an example of a RealTerminal connected to the DUT.

**Electrical Measurements After Programming Bootloader and Firmware**

Measure and record by serial number the following electrical parameters.

Observe the current on the DUT. Press the Mute Switch S601 and the white LEDs D201-D205 should light. The Buzzer will make a sound. Record this measured, full-power current in Table 2 above.

*End of Rev2 Tests as of March 2023.*

**Fig. 19:**
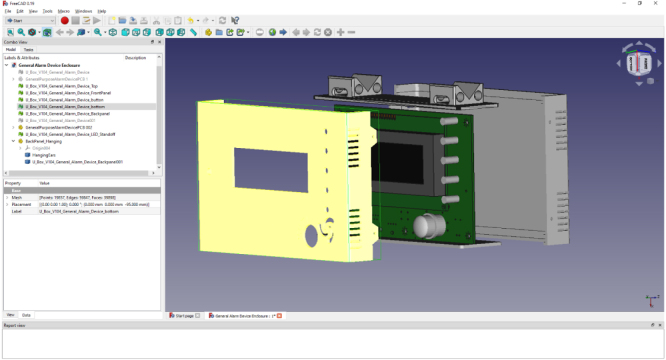
Exploded view of Enclosure Assembly.

**Enclosure Assembly (Refer to** Fig. 19 **)**

Use five screws to hold the printed wiring assembly (Green) to the yellow part. The screws must be selected to fit to the enclosure as 3D printed. The enclosures are printed of nylon and work best with a sheet metal screw of thread diameter 0.14${}^{\prime \prime }$.

Use four screws to fix the grey part to the yellow part, two on each side. The screws must be selected to fit to the enclosure as 3D printed. The enclosures printed of nylon worked with a sheet metal screw of thread diameter 0.087${}^{\prime \prime }$.

## 6 Operation instructions

**How to Control the GPAD as an API**

**Fig. 20:**
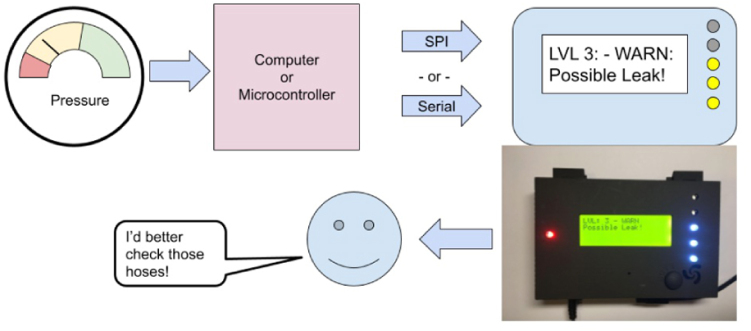
General Usage Process.

This video demonstrated [10] the general usage of the GPAD as depicted in the abstract Fig. 20.

One can use a serial interface through a laptop computer serial monitor to test the GPAD or develop its software. We have designed a trivial interface of commands that the GPAD listens for on the serial interface. Each command begins with one of 4 characters and is terminated by a newline. These are:

• a is the most important command and is used to set the alarm level. The “a” command is of the aN, where N is one of {0,1,2,3,4,5}, specifying increasing alarm levels respectively. These two characters are followed by up to 80 characters defining a message, terminated by an end-of-line (eoln) character.
• h prints a helpful message.
• s means “silence”–mute the GPAD.
• u means “unsilence” or “unmute”–the GPAD is allowed to make noise.

The simplest way to demonstrate the GPAD is by typing commands into the serial port on a computer that acts as a USB host. These commands are of the form:

aNmessage

where “a” is just the character “a” for alarm, “N” is a digit 0, 1, 2, 3, 4 or 5 representing the alarm level, and “message” is a message up to 80 characters long to be displayed.

To become familiar with the API, send these serial commands with a terminal program, or the “serial monitor” featuring the Arduino IDE, which is useful for testing. However, a more typical expected use case is to drive the GPAD with a microcontroller that cannot act as a USB host, and so must use the SPI interface.

Conceptually, the sensing device treats the GPAD as an API—an Application Programmers Interface. The six alarm levels are conceptually named:

• Silent,
• Informational,
• Problem,
• Warning,
• Critical,
• Panic.

The exact meaning of the six levels can be defined by the user (controller) for the application. Of course, not all six must be used. However, the effect of the device should be clear: increasing severity leads to increasing sound and light levels produced by the device to obtain the attention of a human being. The GPAD has 5 LEDs, which are normally unlit in the Silent mode. Each increase in alarm level increases the number of LEDs lit. Our intention is that even from across a room a user may comprehend the alarm level at a glance.

Similarly, at increasing urgency levels, the GPAD plays a different rhythmic pattern of high-pitched sirens, which could be described as a “squeal” or a “scream”. This sound currently gets more urgent-sounding in terms of speed, rhythm, and frequency alternation as alarm level increases. Of course, the user is free to change or improve the coding of the GPAD itself, and a programmer will easily see where we implement the “songs”.

To actually send the message, the programmer of the sensing device will likely use our software library. A C/C++ programmer will understand the API from its actual migration in the file alarm_api.h:



The API has two entry points: alarm and alarm_event. The function alarm is simply a convenience function for calling AlarmEvent which constructs an AlarmEvent for the programmer. An AlarmEvent, as stated above, consists of an AlarmLevel and an 80-character (null-terminated) character array holding the message to be displayed on the LCD.

## Hardware connection

**Fig. 21:**
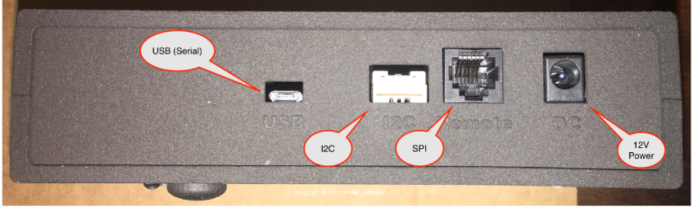
External Ports on Side (Labelled).

The GPAD can be controlled through a USB connection or a SPI connection, as shown in Fig. 21. The SPI connection is made with a RJ12 jack, which was chosen for its ubiquitous availability. (NOTE: The controller can provide 12 V power to the GPAD, or it will be powered through the standard 12 V barrel jack standard 5.5 mm OD 2.1 mm pin.) Often, this will be done with a “wall wart” supply. The GPAD draws less that 1000 mA, even at the highest alarm level.

Schematics in PDF form can be found in the folder at: https://zenodo.org/records/13825787/preview/general-purpose-alarm-device-2.0.2.zip?include_deleted=0#tree_item237.

You can view schematics on-line with the links at: https://zenodo.org/records/13825787/preview/general-purpose-alarm-device-2.0.2.zip?include_deleted=0#tree_item177.

The GPAD requires a 12 V power supply via a standard 5.5 mm OD 2.1 mm pin. It draws less that 1000 mA, even at the highest alarm level.

***SPI Peripheral Pins***

The connector is a JR12 six-position, six-connector (6P6C), and pin 1 is on the left. The connector is held with the contact pins up and facing the observer.

Note the GPAD is a 5 V logic level peripheral device. Level shifters must be used when controlled by a 3.3 V device (see Table 3).

Unfortunately, this format is not compatible with standard breadboards. In experimentation, you may wish to use male-to-female jumper wires.

**Table 3:** SPI peripheral pins.

Pin #	Signal name	Note
1	nCS	Active low
2	COPI	Input to GPAD
3	GND	
4	GLK/SCK	Input to GPAD
5	ControllerVcc	12V Volts, an optional way to put power into the GPAD
6	CIPO	Output from GPAD, 5V.

**I2C Connector**

**Fig. 22:**
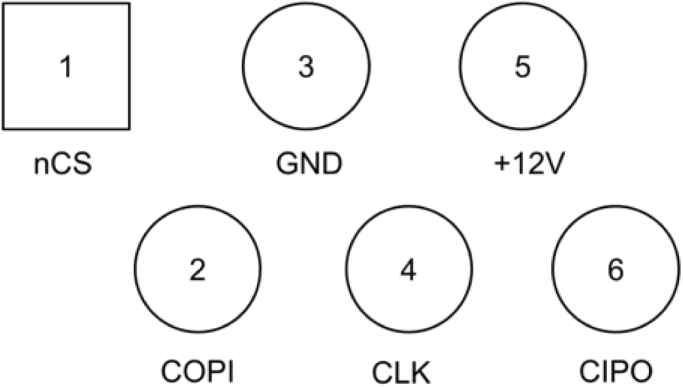
RJ12 PCB Footprint with signal names.

As shown in Fig. 21, there is also an I2C interface to the GPAD.

**Muting**

The GPAD has a mute button. The button silences the sound but does not change the LED lighting. The GPAD stores an internal state as muted or not. Pressing the button toggles the “muted/unmuted” state.

After some experimentation to obtain loudness, we built Revision 2 with two different buzzers (Digi-key part numbers 433-WST-1210T-ND and 102-CMI-1295-0585T-ND) which are both rated at 85 dB at 10 cm. The LEDs are fairly bright. The basic electrical performance of this simple device seems clear.

## 7 Validation and characterization

An online demonstration [11] of the basic functionality of the GPAD is available. This simulates the use of the GPAD with a serial port from a device that is a USB host. This is very convenient for testing, but is probably not the intended use of the GPAD.

The most important use-case for the GPAD is to be controlled by another microcontroller. The SPI interface is particularly useful for controlling a GPAD from another Arduino-class microcontroller. In fact, to make testing as easy as possible, we have software that allows a GPAD peripheral to be controlled by another GPAD through the API. This can be found in the directory GPAD_API_SPI_CONTROLLER (https://zenodo.org/records/13825787/preview/general-purpose-alarm-device-2.0.2.zip?include_deleted=0#tree_item27). This code is a useful starting point for anyone developing a GPAD for any given use case because it exercises the SPI interface, and in particular, the GPAD Application Programmers Interface.

**Fig. 23:**
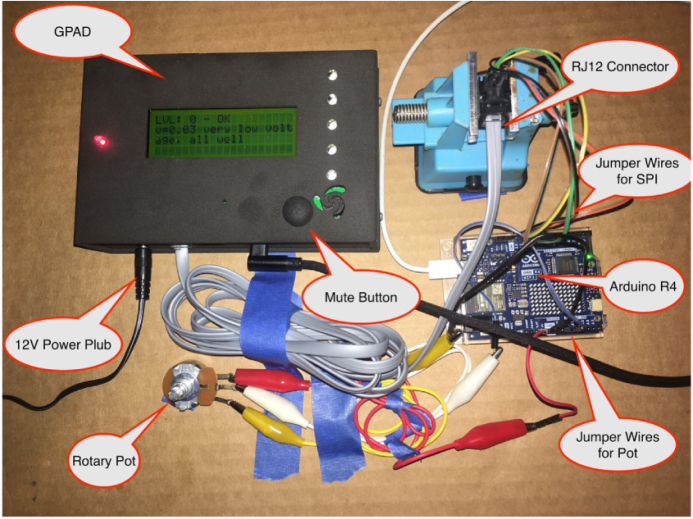
A setup for use testing the GPAD with an Arduino R4.

**Fig. 24:**
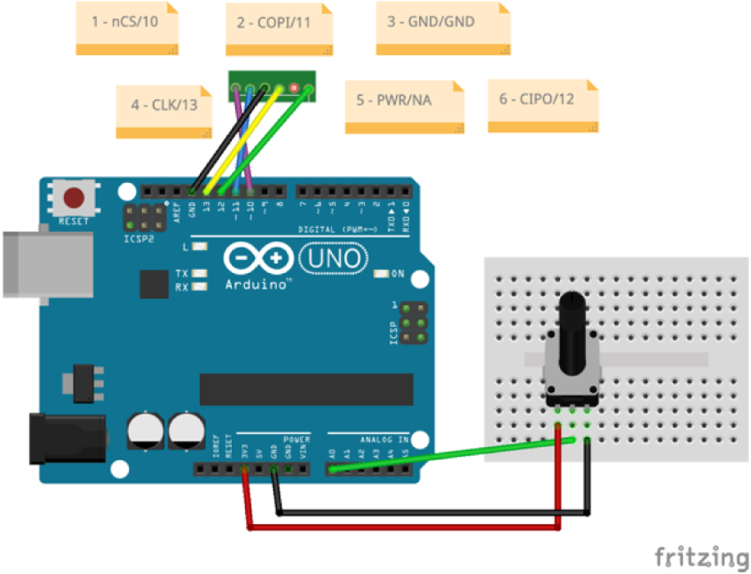
A Fritzing diagram of Arduino Uno as a controller.

However, since it uses a second GPAD as a controller, most users may prefer to use the example described in the video demonstration, which shows how to use an Arduino Uno. The setup of this video is depicted in Fig. 23, and a Fritzing diagram of the circuit is shown in Fig. 24. Please watch our 4:12 min video demonstrating [10] the use of the GPAD with an Arduino Uno before reading this section. This video demonstrates an example circuit and example code available here: https://zenodo.org/records/13825787/preview/general-purpose-alarm-device-2.0.2.zip?include_deleted=0#tree_item4.

This circuit and code are meant to validate the full range of features of the GPAD. Although it uses a simple voltage read of a rotary potentiometer which can be conveniently turned by hand, it is meant to demonstrate the generality of the GPAD, which can be activated by any condition a microcontroller detects or computes.

Note that in Fig. 24 we represent the six-position, six-contract (6P6C) plug as a straight-line header. This plug accepts the RJ12 connector described in the Operating Instructions section (see Fig. 22 and Table 3 above), and should be wired that way, whether you use an RJ12 connector or a direct wiring to an RJ12 cable. (Note: RJ12 connectors are very similar to RJ11 wires, but we need the to use the RJ12 wires.)

**Fig. 25:**
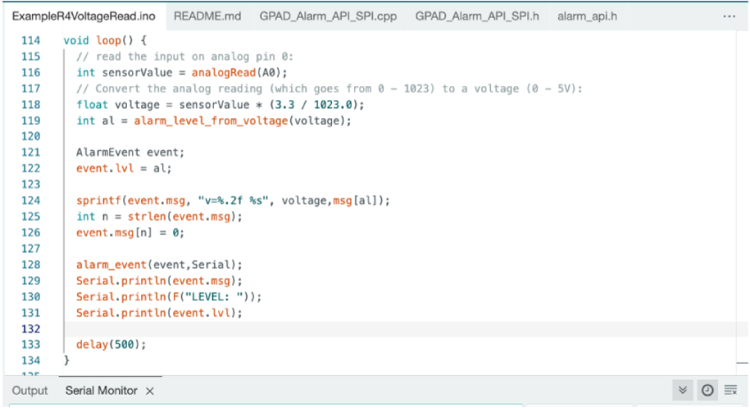
Screenshot of Controller Main Loop.

The rotary potentiometer (pot) is provided with 5 V power and GND. The wiper of the pot is tied to the A0 analog input pin. After adjusting for the maximum and minimum voltage on the wiper, the code shown below breaks the voltage range into intervals associated with the 6 alarm levels (see Fig. 25):

It shows the use of the GPAD_API in the call to the entry point “alarm_event”. It further demonstrates the use of a text message (up to 80 characters), by reporting the voltage (whether it changes the alarm level or not) in every message.

## 8 Conclusion

The GPAD was motivated by the need to alert clinicians to problems with the PolyVent ventilator or the patient, which can be computed by the PolyVent microcontroller. However, it was intentionally designed to be as general purpose as possible. The GPAD could be a tool for making scientific, or other instruments that need to catch human attention.

The GPAD has been demoed in public, including at the 2023 annual OSHWA convention. It has been shown to be loud, bright, and easy to use.

However, the true value of an alarm device can only be measured by its ability to draw human attention, inform a human being how to take action, and not distract them further. There are both simple and complex human/machine interaction performance metrics which we have not begun to measure for the GPAD. A good introduction to the complexity of this field can be found in *Konkani, Oakley and Bauld (2012)* [12]. An example of the continued research into this complex area of human factors research is Edworthy, Parker and Martin [13].

In the language of the field, the GPAD is not yet a “smart alarm” or an “alarm management system”. It is instead an annunciation device (if we called it that, however, non-experts would not understand its intended use). We hope researchers in this area will find the GPAD a useful, programmable component for their systems and investigations.

## CRediT authorship contribution statement

**Robert L. Read:** Writing – review & editing, Writing – original draft, Supervision, Software, Project administration, Methodology, Funding acquisition, Conceptualization. **Lawrence Kincheloe:** Validation, Resources, Investigation. **Forrest Lee Erickson:** Writing – original draft, Validation, Supervision, Resources, Project administration, Methodology, Investigation, Conceptualization.

## Declaration of competing interest

The authors declare that they have no known competing financial interests or personal relationships that could have appeared to influence the work reported in this paper.
